# Thrombotic Role of Blood and Endothelial Cells in Uremia through Phosphatidylserine Exposure and Microparticle Release

**DOI:** 10.1371/journal.pone.0142835

**Published:** 2015-11-16

**Authors:** Chunyan Gao, Rui Xie, Chengyuan Yu, Ruishuang Ma, Weijun Dong, Huan Meng, Yan Zhang, Yu Si, Zhuo Zhang, Valerie Novakovic, Yong Zhang, Junjie Kou, Yayan Bi, Baoxin Li, Rujuan Xie, Gary E. Gilbert, Jin Zhou, Jialan Shi

**Affiliations:** 1 Department of Hematology, The First Hospital, Harbin Medical University, Harbin, China; 2 Department of Medical Laboratory Science and Technology, Harbin Medical University-Daqing, Daqing, China; 3 Department of Gastrointestinal Oncology, The Third Hospital, Harbin Medical University, Harbin, China; 4 Department of Nephrology, The First Hospital, Harbin Medical University, Harbin, China; 5 Department of general surgery, The fifth Hospital, Harbin Medical University, Harbin, China; 6 Department of Cardiology, The Second Hospital, Harbin Medical University, Harbin, China; 7 Department of Pharmacology, Harbin Medical University, Harbin, China; 8 Medicine Departments, VA Boston Healthcare System, Brigham and Women’s Hospital, Harvard Medical School, Boston, MA, United States of America; INSERM, FRANCE

## Abstract

The mechanisms contributing to an increased risk of thrombosis in uremia are complex and require clarification. There is scant morphological evidence of membrane-dependent binding of factor Xa (FXa) and factor Va (FVa) on endothelial cells (EC) *in vitro*. Our objectives were to confirm that exposed phosphatidylserine (PS) on microparticle (MP), EC, and peripheral blood cell (PBC) has a prothrombotic role in uremic patients and to provide visible and morphological evidence of PS-dependent prothrombinase assembly *in vitro*. We found that uremic patients had more circulating MP (derived from PBC and EC) than controls. Additionally, patients had more exposed PS on their MPs and PBCs, especially in the hemodialysis group. *In vitro*, EC exposed more PS in uremic toxins or serum. Moreover, reconstitution experiments showed that at the early stages, PS exposure was partially reversible. Using confocal microscopy, we observed that PS-rich membranes of EC and MP provided binding sites for FVa and FXa. Further, exposure of PS in uremia resulted in increased generation of FXa, thrombin, and fibrin and significantly shortened coagulation time. Lactadherin, a protein that blocks PS, reduced 80% of procoagulant activity on PBC, EC, and MP. Our results suggest that PBC and EC in uremic milieu are easily injured or activated, which exposes PS and causes a release of MP, providing abundant procoagulant membrane surfaces and thus facilitating thrombus formation. Blocking PS binding sites could become a new therapeutic target for preventing thrombosis.

## Introduction

Uremic patients are at high risk of both venous and arterial thrombosis, and this may represent the predominant cause of mortality especial in hemodialysis (HD) [1–3]. Although this thrombophilia is considered to be multi-factorial [4], the mechanisms specific to uremia that promote a hypercoagulable state have not yet been identified. Furthermore, delivering appropriate antithrombotic therapy to uremic patients that balances safety and efficacy remains a challenge [5,6]. This has triggered research efforts to elucidate the pathogenesis of thrombotic tendencies in uremia and to provide feasible therapeutic strategies to reduce mortality associated with these complications.

The pathogenesis of hypercoagulable state in uremia includes increased procoagulant factors, down-regulation of anticoagulant function, hyperfibrinogenemia, impaired fibrinolysis, and elevated levels of inflammatory mediators during chronic or recurrent uremic inflammation [7–10]. However, besides these plasmatic abnormalities, peripheral blood cells (PBCs) and endothelial cells (ECs) are a major concern because they are in constant contact with uremic retention solutes, increasing the potential for injury or activation by uremic toxins. This is of particular concern for those patients on dialysis regimens.

Phosphatidylserine (PS), one of the four major phospholipids, is usually confined to the inner leaflet of the cell membrane. During cell apoptosis or activation, PS is exposed on the outer membrane [11]. Activated cells also release vesicles, called microparticles (MPs), which expose PS and express membrane antigens on their surfaces [12]. The exposure of PS on cells and MPs provides binding sites for factor Xase (FXa) and prothrombinase complexes and thereby catalyses the coagulation cascade [13]. Several studies showed that increased PS externalization in erythrocytes and platelets may play a role in the thrombotic risk of uremia [14,15], however, it is not entirely clear whether ECs, leukocytes (including polymorphonuclear cell (PMN) and mononuclear cell (MNC)) contribute to uremic hypercoagulable state, and how these cell types affect hemostatic balance within the uremic milieu. We postulate that accelerated damage or activation of blood cells and ECs in uremic milieu may contribute to the thrombogenesis through PS exposure and MP release. Lactadherin, a sensitive probe for PS exposure and an anticoagulant that competes with factors V and VIII for membrane-binding sites [16–19], was used to detect and block procoagulant PS on PBCs of uremic patients or EC cultured with uremic serum or mixed toxins.

In this study, an integrated assessment of uremia-enhanced thrombogenicity was performed. We measured the amount of PS exposure and MP release on RBC, platelet, PMN, and MNC in uremic patients and on EC incubated in medium that mimicked the *in vivo* conditions. We used site-specifically labeled fluorescent species of FVa and FXa to image the distribution of prothrombinase. Finally, we examined the PS-dependent contribution of MPs, blood cells, and ECs to hypercoagulability in uremic patients.

## Materials and Methods

### Reagents

Human umbilical vein endothelial cells (HUVECs) and ECs medium were from ScienCell (San Diego, CA, USA). Trypsin-EDTA was from Gibco. Urea, creatine, oxalate, uric acid, indoxyl sulfate, p-cresol (4-methylphenol), homocysteine and indole-3-acetic acid were obtained from Sigma (Saint Quentin Fallavier, France). Megamix beads (mix of 0.5, 0.9 and 3.0 μm beads) were from Biocytex (Marseille, France). Trucount Tube (Cat. No. 340334), Annexin V, Propidium iodide (PI), purified CD235a (clone GA-R2), CD31 (clone L133.1), CD41a (clone HIP8), CD45 (clone 2D1), CD66b (clone G10F5), CD14 (clone M5E2), CD3 (clone 1F4), CD142 (clone HFT-1), and mouse IgG1/IgG2a (clone X40/X39) were from Becton Dickinson Biosciences (San Jose, CA, USA). All these monoclonal antibodies were labeled in our laboratory with Alexa Fluor 647 or Alexa Fluor 488 (Invitrogen). Alexa Fluor 488 or Alexa Fluor 647-conjugated lactadherin and fibrin were prepared in our laboratory. Human factors Va, VIIa, IXa, X, Xa, prothrombin, thrombin, fluorescein EGR-Chloromethylketone, and biotinylated EGR-Chloromethylketone were all from Haematologic Technologies (Burlington, VT, USA). Recombinant human factor VIII was from American Diagnostica Inc. Mouse anti-fibrin II β chain (clone NYBT2G1) was from Accurate Chemical & Scientific (Westbury, NY, USA). Fluorescein-maleimide, fluorescein isothiocyanate phalloidin, DAPI, Alexa 647–labeled isotype-matched control antibody were from Molecular Probes (Invitrogen, Eugene, OR, USA). Percoll were from GE Healthcare (Uppsala, Sweden). Ficoll-Hypaque were from Sigma-Aldrich (St Louis, MO, USA). Chromogenic substrates S-2765 and S-2238 were from Instrumentation Laboratory Company (MA, USA). Tyrode’s buffer containing 1 mM Hepes was from our laboratory and was filtered through a 0.22-μm syringe filter from Millipore (UK).

### Patients

This study was performed with written informed consent from each participant and approval from the Ethics Committee of Harbin Medical University according to the Helsinki Declaration. Uremic patients including 25 non-dialysis (Non-D), 18 continuous ambulatory peritoneal dialysis (CAPD), and 23 on haemodialysis (HD) were studied between July 2010 and September 2013. The cause of renal failure was nephroangiosclerosis (n = 14), chronic glomerulonephritis (n = 27), chronic interstitial nephritis (n = 19), polycystic kidney disease (n = 2), and undetermined (n = 4). The Non-D patients had a decreased kidney glomerular filtration rate (GFR) of < 30 mL·min·1.73 m^-2^ for ≥3 months. All CAPD patients had four exchanges daily of 2 L of dialysate (1.5 to 4.25% dextrose). All HD patients were being dialyzed three times a week with a four-hour dialysis session using bicarbonate dialysate using heparin as an anticoagulant at an infusion rate of 1000 U/h. Blood flow rate was 280 ml/min and the dialysate (bicarbonate) flow rate was 500 ml/min. Ultrafiltration varied according to the patient’s actual weight. Exclusion criteria included: diabetes mellitus; malignant or systemic disease; iron, folic acid, and vitamin B12 deficiency; blood transfusion within the past six months; uncontrolled hypertension; active or chronic infection; a Kt/V ratio of < 1.2; and any drug known to affect haemostasis (except for heparin during HD procedure). A majority of patients had been treated with recombinant human erythropoietin (rHuEPO) and antihypertensive medications (angiotensin converting enzyme inhibitors or calcium antagonists) at a stabilized dosage. Twenty healthy subjects were included as parallel controls. The subjects’ profiles are shown in Table 1.

**Table 1:** Baseline characteristics of patients with uremia and healthy subjects at inclusion.

		Controls	Non-D	CAPD	HD
**Sex (male/female)**		**11/9**	**14/11**	**9/9**	**11/12**
**Age (years)**		**52.9±11.8**	**49.6±14.3**	**53.4±8.4**	**52.6±14.1**
**Duration of dialysis (years)**		**–**	**–**	**1.9±0.9**	**2.4±1.5**
**RBC count (10** ^**12**^ **/L)**		**4.4±0.4**	**2.7±0.5** ***	**2.6±0.6** ***	**2.8±0.5** ***
**Hemoglobin (g/L)**		**132.2±11.4**	**109.8±8.3** ***	**108.8±10.3** ***	**105.2±7.3** ***
**Platelet count (10** ^**9**^ **/L)**		**200.8±53.2**	**190.9±43.9**	**199.4±80.0**	**203.5±47.3**
**WBC count (10** ^**9**^ **/L)**		**6.1±1.3**	**6.8±1.7**	**7.3±2.4** *	**7.2±1.9**
**Creatinine (μmol/L)**		**70.3±10.4**	**1195.8±524** ***	**872.5±256.2** *** ^##^	**843.3±138.5** *** ^###^
**BUN (mmol/L)**		**6.9±2.3**	**30.5±7.4** ***	**19.6±6.2** *** ^###^	**22.5±7.6** *** ^###^
**PT (s)**		**11.7±1.0**	**11.7±0.9**	**11.8±0.9**	**11.6±0.9**
**APTT (s)**		**32.2±4.5**	**26.0±3.3** ***	**26.4±2.2** ***	**25.9±3.6** ***
**Fibrinogen (g/L)**		**3.5±0.9**	**3.9±0.7**	**3.4±0.7**	**3.7±0.7**
**D-dimer (ng/ml)**		**98.4±49.0**	**191.2±90.0** ***	**173.7±82.8** **	**185.7±82.6** ***
**Mean SBP(mmHg)**		**129.3±8.4**	**134±10.5**	**131.7±8.9**	**135.4±8.7**
**Mean DBP(mmHg)**		**75.4±6.4**	**78.4±5.8**	**78.6±5.5**	**79.2±5.9**
**Treatment (%)**	**ACE inhibitors**	**–**	**38%**	**35%**	**37%**
	**Calcium antagonists**	**–**	**20%**	**18%**	**22%**
**Cardiovascular events**		**0**	**2**	**1**	**6**

Non-D, non-dialyzed; CAPD: continuous ambulatory peritoneal dialysis; HD, hemodialysis; BUN, blood urea nitrogen; SBP: systolic blood pressure; DBP: diastolicblood pressure; ACE, angiotensin converting enzyme. Data are means ± SD,

*P < 0.05,

**P < 0.01,

***P < 0.001 versus controls.

^##^P < 0.01,

^###^P < 0.001 versus Non-D.

### Protein purification and labeling

Bovine lactadherin was purified as previously described, and was labeled with Alexa Fluor 488 or Alexa Fluor 647 according to the package instructions. The ratio of fluorescein to lactadherin was 1.2/1 and 1.1/1, respectively [20,21].

### Preparation of blood cells and MPs

Blood samples were drawn by antecubital venipuncture and anticoagulated with trisodium citrate 0.109 mol/L. RBC and platelet-rich plasma (PRP) were prepared within 30 min after blood collection by centrifugation for 15 min at 200 x g at room temperature. PRP were centrifuged 20 min at 1500 g, and plasma was then harvested and centrifuged 2 min at 13 000 g to remove all residual platelets to obtain platelet-free plasma (PFP). PFP samples were snap-frozen in liquid nitrogen, and then stored at −80°C until use. In order to isolate the MPs, 250 μl of PFP was thawed on ice for 60 min and then centrifuged for 45 min at 106 000 g at 4°C. Subsequently, 225 μl of supernatant (i.e. MP-depleted plasma) were removed. The remaining 25 μl MPs pellet was washed once and resuspended in 75 μl of Tyrode’s buffer.

Polymorphonuclear cell (PMN) were immediately separated by means of differential centrifugation gradient with Percoll as our previously described [22]. In brief, 40 ml of fresh venous blood was drawn from each uremic patient and healthy subject, and blended lightly in four 15-ml polypropylene conical tubes containing 200 μl of 100 mM ethylenediaminetetraacetic acid (EDTA). After centrifuged at 200 x g for 15min at 20°C, PRP was aspirated carefully and centrifuged again at 1,500 x g for 20 min. The resulting platelet-poor plasma (PPP) was collected in a 15-ml tube. Subsequently, 3 ml of 6% dextran (500,000 mol wt; Amersham Bios- ciences, Uppsala, Sweden) was added and the total volume was brought up to 15 ml with 0.9% sodium chloride solution. The mixture was set aside for erythrocyte sedimentation for 30 min at room temperature. The leukocyte-rich and erythrocyte-poor upper layer was collected and centrifuged at 300 x g for 5 min. The pellet was resuspended with 2 ml of PPP and transferred to the tube containing layered Percoll (2 ml of 42% Percoll on top of 2 ml of 51% Percoll). The sample was then centrifuged at 300 x g for 20 min at 20°C, and the PMNs were aspirated from the interface between the 51% and 42% Percoll layers. After washing twice with Hanks balanced salt solution (HBSS, without Ca^2+^ and Mg^2+^) (Thermo Fisher HyClone, Logan, UT, USA), the concentration was determined by manual counting with a hemacytometer.

Mononuclear cells (MNC) were purified by density gradient centrifugation using Ficoll-Hypaque, washed twice with Tyrode’s buffer, and then used for labeling.

### Preparation of plasma and serum

For MP-depleted plasma (MDP) preparation, PRP was centrifuged 20 min at 1500 g, and then the supernatant was spun for 2 min at 13 000 g. The resulting platelet free plasma (PFP) was centrifuged at 106 000 g for 1 hour at 4°C to remove MPs, aliquoted, and stored at -80°C. For serum preparation, blood was collected in vacutainer tubes and separated by centrifuging clotted blood at 1100 g for 10 minutes at room temperature.

### Preparation of mixed toxins

Urea, creatinine, oxalic acid, IA, IS, and homocysteine were dissolved in water. UA being water insoluble was dissolved in 1M NaOH and p-cresol was dissolved in DMSO.

### Flow cytometric analysis MPs phenotype

MPs were identified as previously reported [23]. Erythrocyte, platelet, pan-leukocyte, granulocyte, lymphocyte, monocyte, and EC-derived MPs were defined as smaller than 1 μm and either lactadherin^+^ CD235a^+^, lactadherin^+^ CD41a^+^, lactadherin^+^ CD45^+^, lactadherin^+^ CD66b^+^, lactadherin^+^ CD3^+^, lactadherin^+^ CD14^+^, or lactadherin^+^ CD31^+^ CD41a^-^, respectively [24]. MPs exposing TF were identified as those with coexpression of lactadherin and CD142. In another experiment, platelet derived MPs (PMPs) were also labelled with annexin V and CD41a. The number of each MP type per μl was calculated by Trucount Tube (with a precise number of fluorescent beads 48678 to determine the number of MPs in a sample) after accumulation of 10,000 gated events.

### Flow cytometric analysis of PS exposure on PBC and EC

To quantify PS exposure, RBCs, platelets, PMNs, MNCs, or ECs suspended in Tyrode’s buffer were adjusted to 0.5–1×10^6^/ml, then 5 μl Alexa Fluor 488-conjugated lactadherin or annexin V was added to the cell suspension and incubated for 10 min at room temperature in the dark. Ten thousand events per sample were acquired and analyzed with BD FACS Diva Software.

### Confocal microscopy

To locate PS, RBCs or WBCs were incubated with the indicated concentrations of Alexa Fluor 488-lactadherin and PI while ECs were incubated with Alexa Fluor 488-lactadherin and Alexa Fluor 647-CD31 for 10 min at room temperature in the dark. Cells were then washed to remove unbound dye and analyzed immediately. Observation of the PS exposure on platelets and MPs by confocal microscopy was carried out as previously described [25]. Images were obtained in LSM 510 SYSTEM (Carl Zeiss Jena GmbH, Jena, Germany).

### Endothelial cell culture and reconstitution experiments

ECs were cultured in EGM-2 medium under standard cell culture conditions (humidified atmosphere, 5% CO_2_, 37°C). To assess the effect of uremic media, ECs were cultured for 48 h in growth media containing mixed uremic toxins (urea, 1200 μg/ml; creatine, 60 μg/ml; oxalic acid, 5 μg/ml; uric acid (UA), 80 μg/ml; p-cresol, 10 μg/ml; indoxyl sulfate (IS), 25 μg/ml; homocysteine, 2.7 μg/ml; indole-3-acetic acid (IA), 3.5 μg/ml at median concentrations observed in uremic patient [26]) or 20% pooled serum obtained from 10 different uremic patients or age-matched healthy donors. At designated time points (every four hours), PS exposure was detected by flow cytometer. In another set of experiments, after incubation for 24 h in uremic media, ECs were harvested and washed twice with EGM-2 medium and then incubated with normal serum. At prescribed time points, cells were analyzed for PS exposure. Each test was performed in triplicate.

### Procoagulant activity and inhibition assays

Procoagulant activity (PCA) of the various cell types and MPs was evaluated using a one-stage recalcification time assay in a KC4A-coagulometer (Amelung, Labcon, Heppenheim, Germany) [23]. For inhibition assays, the cell or MP suspensions were preincubated with lactadherin at a final concentration of 128 nM.

### FXa and prothrombinase assays

The formation of intrinsic FXa in the presence of cells was performed as follows. A total of 10^5^ RBCs, 10^4^ other cell types, or 10 μl of MPs-enriched suspension was incubated with 1 nM FIXa, 130 nM FX, 5 nM FVIII, 0.2 nM thrombin and 5 mM Ca^2+^ in FXa buffer for 5 min at 25°C. The reaction was stopped by addition of EDTA at a final concentration of 7 mM. Immediately after the addition of 10 μl S-2765 (0.8 mM) to each reaction, the quantity of FXa formed was measured as absorbance at 405 nm on a Universal Microplate Spectrophotometer (PowerWave XS; Bio-Tek) in kinetic mode. Absorbance results were converted to concentration using a standard dilution of FXa. Measurement of extrinsic FXa formation was analogous to that for intrinsic FXa except that cells were incubated with 130 nM FX, 1 nM FVIIa and 5 mM Ca^2+^. For the prothrombinase assay, cells were incubated with 1 nM FVa, 0.05 nM FXa, 1 μM prothrombin and 5 mM Ca^2+^ in prothrombinase buffer for 5 min at 25°C. EDTA and S-2238 were added to each microplate, and thrombin production was assessed as previously described [23]. To test the inhibition of the coagulation complexes by lactadherin, each kind of cell was preincubated with 128 nM lactadherin for 10 min at 25°C in Tyrode’s buffer. The quantity of thrombin or FXa formation was then assessed as above.

### FVa/FXa binding and fibrin formation on cultured endothelial cell and circulating MPs

To observe FVa and Xa binding, ECs or MPs were co-stained with factor Va-fluorescein-maleimide and factor Xa-EGRck-biotin (complexed to Alexa 647-steptavidin). For *in vitro* fibrin generation experiments, ECs were cultured on a gelatin-coated coverslip chamber with 20% pooled uremic or normal serum for 24h. Cells were then rinsed with Tyrode’s buffer, and overlaid with prewarmed MDP (15%) in the presence of 3 mM calcium. To observe the contribution of MPs to fibrin formation, MPs-containing suspensions were incubated with prewarmed MDP (15%) in the presence of 3 mM calcium. Fibrin networks were imaged using laser confocal microscopy in the presence of Alexa Fluor 647-conjugated anti-fibrin. Background signal was calculated using a similarly labeled, isotype-matched control antibody. Cells and nuclei were visualized by actin staining with fluorescein isothiocyanate phalloidin (1 μg/ml) and DAPI (4’,6-diamidino-2-phenylindole; 300 nM), respectively. MPs were stained with Alexa fluor 488 -labeled lactadherin and Alexa fluor 647 -labeled Annexin V. Fibrin formation was quantified by turbidity as described [27]. Isolated MPs or cultured ECs were added to re-calcified (10 mM, final) MDP (88% MDP, final) in the absence or presence of 128 nM lactadherin. Fibrin formation was measured by turbidity at 405 nm in a SpectraMax 340PC plate reader.

### Statistical analysis

Numerical variables were tested for normal distribution with the Kolmogorov-Smirnov test. Data are expressed as mean ± standard deviation (SD), and statistical analysis was made by t-test or ANOVA as appropriate. Categorical variables were compared using the χ^2^-test. Linear regression analysis was used to detect any relation between EMP levels and serum creatinine and BUN. P < 0.05 was considered statistically significant.

## Results

### Subject Characteristics

Clinical characteristics of uremic patients and controls are shown in Table 1. Uremic patients had significantly higher creatinine, BUN, shortened aPTT, lower RBC count and hemoglobin level. Furthermore, creatinine and BUN in HD patients was lower than that in Non-D, while there was a higher number of thromboembolic events in patients with HD (P < 0.05 across groups).

### Number and origin of MPs in patients with uremia

**Fig 1:**
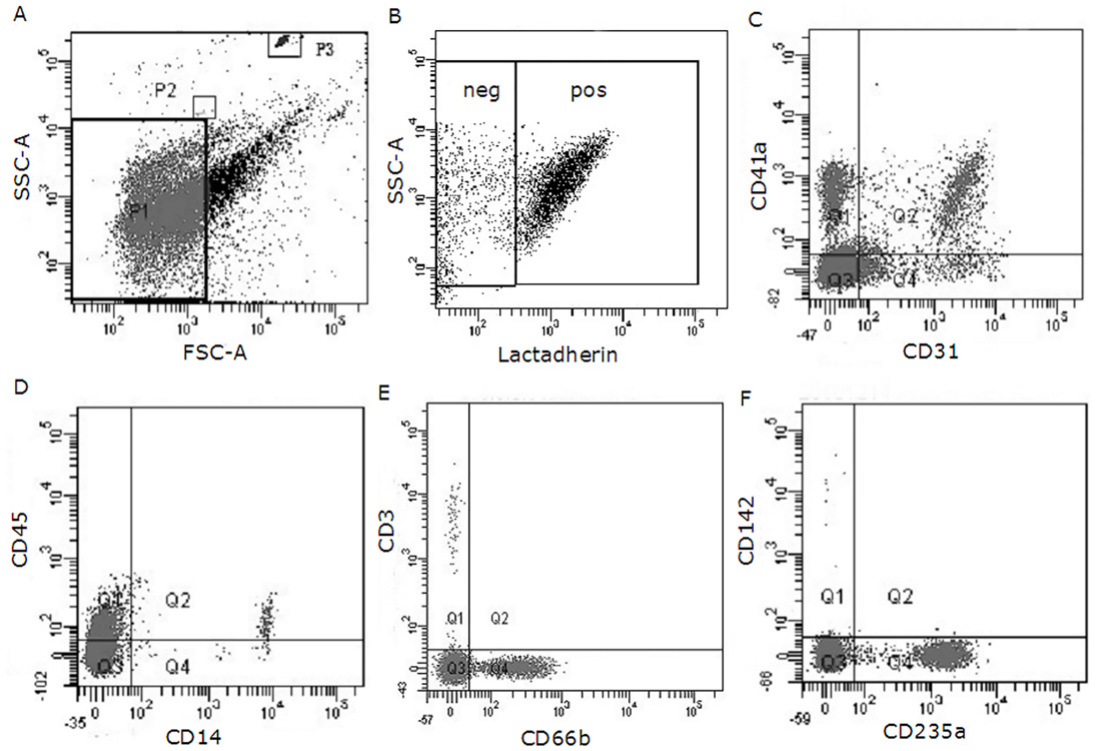
Flow cytometry analyses of MPs in a sample from a HD patient. A representative set of scatter grams in a sample from a HD patient is shown to illustrate MPs and subpopulation definition. Results were acquired and analyzed with BD FACS Diva Software. (A) Forward and side scatter were used to define the events with a size of smaller than 1 μm which were gated in the P1 window. P2 and P3 are drawn around 1.0-μm and Trucount beads, respectively. (B) Events were then selected for their lactadherin binding, determined by positivity for lactadherin-Alexa Fluor 488 (on the x-axis). (C) Lactadherin-positive MPs were further examined for expression of other antigens by co-labeling with Alexa Fluor 488- and Alexa Fluor 647-labeled antibodies as is shown here for PMPs (Alexa Fluro 647-CD41a^+^) and EMPs (Alexa Fluro 488-CD31^+^/ Alexa Fluor 647-CD41a^-^). (D) LMPs (pan-leukocyte, Alexa Fluor 647-CD45^+^) and monocyte origin -MPs (Alexa Fluor-488 CD14^+^). (E) MPs derived from lymphocyte (Alexa Fluro 647-CD3^+^) and neutrophil (Alexa Fluor 488-CD66b^+^). (F) RMPs (Alexa Fluor 488-CD235a^+^) and the TF expressing MPs (Alexa Fluor 647-CD142^+^). HD, haemodialysis; MP, microparticle; PMPs, platelet MPs; EMPs, endothelial cell MPs; LMPs, leukocyte MPs; RMPs, RBC MPs; TF, tissue factor; neg, negative; pos, positive.

We first tested the total number of MPs and their phenotypic characteristics in healthy subjects and uremic patients. TruCount beads acted as an internal standard and enabled us to calculate the absolute number of MPs per volume of specimen (Fig 1A). For most study subjects, more than 90% of gated events bound to lactadherin, indicating PS exposure (Fig 1B). MP derived from platelets (CD41a^+^ PMP), endothelial cells (CD31^+^/41^-^ EMP), pan-leukocytes (CD45^+^ LMP), mononuclear cell (CD14^+^), T lymphocyte (CD3^+^), granulocyte (CD66b^+^), erythrocytes (CD235a^+^ RMP), and TF (tissue factor, CD142^+^) are shown in Fig 1C and 1F. The total number of MPs (lactadherin^**+**^ MP; P< 0.001) was higher in uremia patients than in healthy subjects, with elevated levels of PMP, RMP, LMP and EMP (P < 0.001) specifically. In addition, HD patients also had significantly more PMPs, RMPs, and LMPs (including T lymphocyte, mononuclear cell, and granulocyte-derived MPs) than CAPD or Non-D patients. No statistical differences in EMP levels were found between CAPD, Non-D, and HD patients (Table 2). However, linear regression analysis showed that the total amount of EMPs was correlated with the level of creatinine (r = 0.527, p < 0.05) (S1 Table). Few MPs with co-expression of both PS and TF could be identified in either uremic patients or healthy controls (Table 2). Double staining with anti-CD41 and lactadherin / annexin V was used to identify PMPs number in uremic patients. In these experiments, lactadherin detected significantly higher numbers of CD41 / PS-positive MPs than did annexin V (Panel A and C in S1 Fig).

**Table 2:** Microparticles per microliter of plasma in Non-D, CAPD, HD and controls.

	Controls	Non-D	CAPD	HD
**Total lactadherin** ^**+**^ **MPs (/μl)**	**1595.3±225.8**	**3685.9±560.1** ***	**3734.1±533.8** ***	**4184.5±621.8** ^#^
**lactadherin** ^**+**^ **CD41a** ^**+**^	**1283.7±228.3**	**3026.6±647.8** ***	**2957.4±372.1** ***	**3681.7±440.7** ^###^
**lactadherin** ^**+**^ **CD235a** ^**+**^	**22.5±4.9**	**214.6±52.0** ***	**221.2±53.9** ***	**298.8±45.6** ^###^
**lactadherin** ^**+**^ **CD31** ^**+**^ **CD41a** ^**-**^	**12.9±3.3**	**149.2±36.8** ***	**164.6±18.7** ***	**163.7±33.6** ***
**lactadherin** ^**+**^ **CD45** ^**+**^	**25.5±10.4**	**81.2±20.5** ***	**80.2±25.6** ***	**103.4±24.7** ^##^
**lactadherin** ^**+**^ **CD66b** ^**+**^	**11.6±4.0**	**55.6±22.7** ***	**52.6±23.8** ***	**80.3±27.2** ^###^
**lactadherin** ^**+**^ **CD14** ^**+**^	**6.8±2.6**	**15.5±5.3** ***	**15.7±4.9** ***	**19.2±6.6** ^#^
**lactadherin** ^**+**^ **CD3** ^**+**^	**4.3±2.3**	**11.1±4.0** ***	**12.4±3.0** ***	**16.9±4.1** ^###^
**lactadherin** ^**+**^ **CD142** ^**+**^	**2.3±1.7**	**2.5±2.0**	**2.5±2.1**	**3.0±2.7**

MP, microparticle; Non-D, non-dialyzed; CAPD: continuous ambulatory peritoneal dialysis; HD, hemodialysis. Corrected for the number of events with isotype controls,

***P < 0.001 versus controls.

^#^P < 0.05,

^##^P < 0.01,

^###^P < 0.001 versus Non-D and CAPD.

### PS exposure of blood cell in healthy subjects and uremic patients

**Fig 2:**
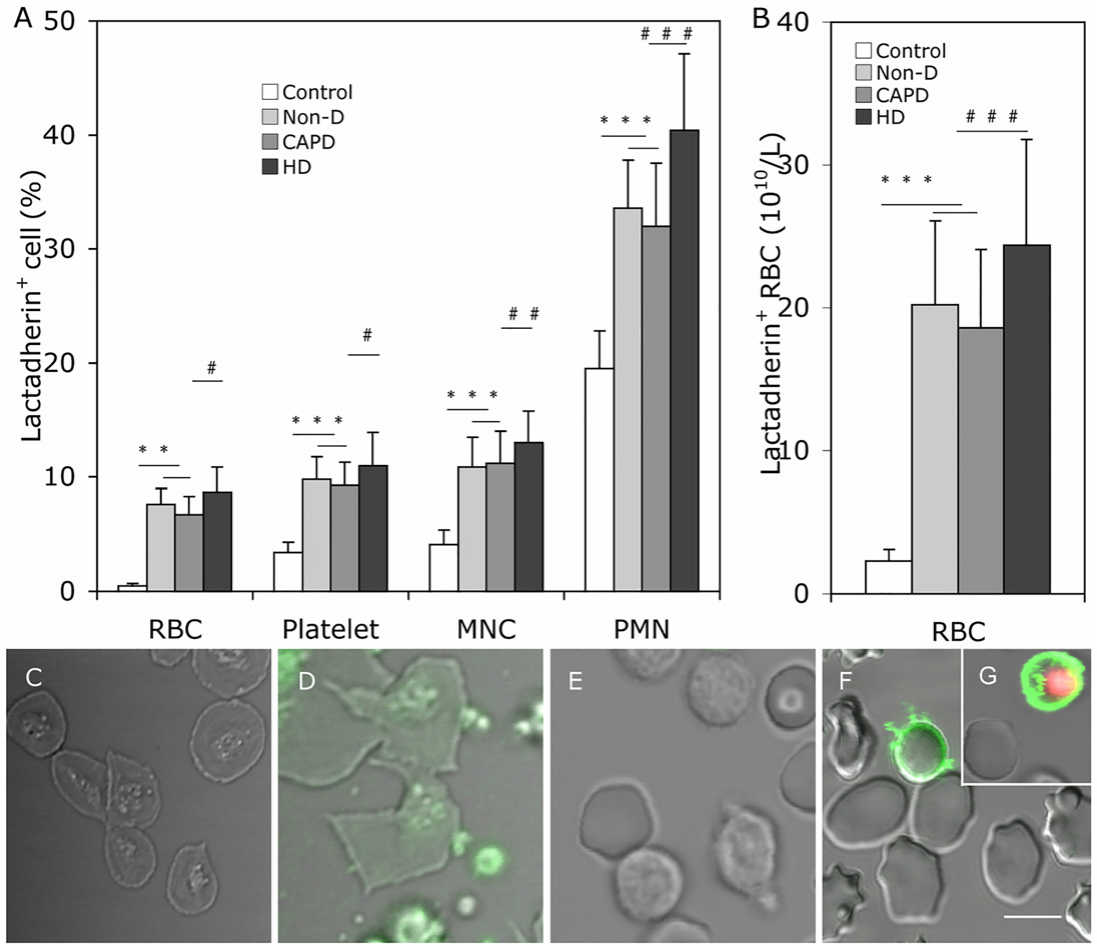
PS exposure on the plasma membrane of blood cells. Comparison of PS exposure on blood cells among Non-D patients, CAPD patients, HD patients and healthy subjects. Cells were incubated with Alexa Fluro 488 -lactadherin separately in the dark for 10 min at room temperature before evaluation by flow cytometry. (**A**) We measured the percent of RBCs, platelets, PMNs, MNCs that bound lactadherin from healthy subjects (n = 20), Non-D (n = 25), CAPD (n = 18) and HD patients (n = 23). (**B**) Lactadherin -binding RBC number per liter of plasma according their count in each person. Data are expressed as mean ± SD (***P < 0.001, ^#^P < 0.05, ^##^P < 0.01, ^###^P < 0.001). PS exposure on the plasma membrane of blood cells was observed by confocal microscopy with LSM 510 3.2 SP2 software. Platelets, RBCs and WBCs of healthy subjects and uremic patients were incubated with Alexa Fluro 488 -lactadherin and PI in the dark 10 min at room temperature. Cells were then washed very gently to remove unbound dye. Cell membrane displayed green fluorescence when labeled with lactadherin and nucleus displayed red fluorescence. Lactadherin staining (green) is observed on platelets membrane and MPs **(D)** and RBC **(F)**/WBC **(G)** in uremic patient but no staining in healthy subjects **(C, E)**. The inset bar equals 5 μm. PS: phosphatidylserine; PMN: polymorphomuclear cell; MNC: mononuclear cell; Non-D: Non-dialysis; CAPD: continuous ambulatory peritoneal dialysis; HD: haemodialysis.

The mean percentage of lactadherin^+^ RBC, platelet, PMN, and MNC in uremic patient was significantly higher than that in healthy subjects, and greater in HD than in CAPD and Non-D patients (P < 0.05 for each kind of cell, Fig 2A). Additionally, the absolute number of lactadherin^+^ RBC in each uremic group was 10 fold higher than the control (Fig 2B). In separate experiments, annexin V was used to determine the percentage of PS exposure on platelets. Comparing the results showed that lactadherin was able to detect higher levels of PS exposure in platelets than annexin V in each group (Panel B and D in S1 Fig).

To observe PS on the outer membrane of cells, RBCs/leukocytes/platelets of a healthy subject and a uremic patient were incubated with Alexa Fluor 488-lactadherin and PI (for leukocytes) and imaged on a confocal microscope. As shown in Fig 2C and 2E, almost no staining by Alexa Fluor 488-lactadherin could be detected on platelet, RBC, and leukocyte membranes in healthy subjects. Lactadherin binding on platelets from uremic patients accompanied vesiculation formation (Fig 2D), and lactadherin staining of RBCs (Fig 2F) and leukocytes (Fig 2G) indicated PS exposure in uremic patients.

### PS exposure of cultured endothelial cells

**Fig 3:**
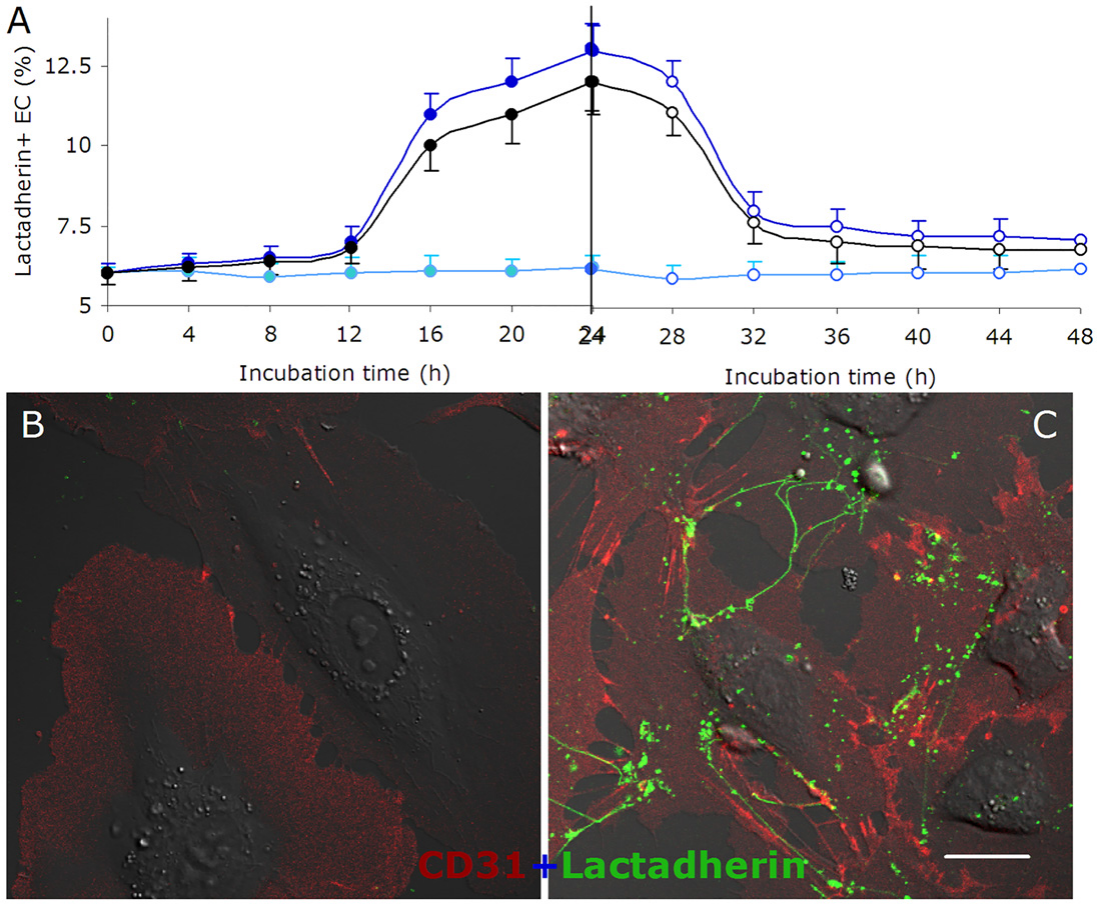
PS exposure and reconstitution experiments of cultured HUVECs. ECs were cultured in medium with normal serum (●, ○), uremic serum (●, ○), and normal serum with mixed uremic toxins (●, ○) for 24h, respectively (filled circles). Then the cells were washed and incubated with normal serum for another 24h (open circles). At indicated time points (0, 4, 8, 12, 16, 20, 24, 28, 32, 36, 40, 44, 48h), ECs were collected and incubated with lactadherin -Alexa Fluor 488 for 10 min in the dark before evaluation by flow cytometry. ECs cultured in normal serum for 48h were used as control. Each point represents mean ± SD for triplicate samples of independent experiments. **(A)** Kinetic mode of PS reversal on EC with different treatments. PS exposure on the outer membrane surface of EC occurred mainly from 12 to 24h when cultured with uremic serum or mixed toxins. After washing ECs, about 80% of PS reverted to the inner leaflet during the first 8h of culture with normal serum. ECs cultured for 24 h with normal or uremic serum were stained with CD31-Alexa Fluor 647 and PS exposure was detected using lactadherin-Alexa Fluor 488 and visualized using confocal microscopy. **(B)** Almost no lactadherin staining was observed on ECs cultured in normal serum. **(C)** Treatment of ECs with uremic serum led to retraction of cell margins, extension of filopods, and lactadherin (green) binding on filopods. The inset bar equals 10 μm.

PS exposure of cultured ECs in uremic serum or mixed toxins increased gradually during the first 12h and rapidly from 12-24h (Fig 3A left part). From 24h to 48h, the proportion of PS exposure on ECs continued to increase slowly in uremic serum or mixed toxins (data not shown), while ECs cultured in normal serum showed almost no changes during 48h. To examine whether PS exposure on EC membrane was reversible, reconstitution experiments were performed. ECs exposed to uremic serum or mixed toxins for 24h were washed and cultured in normal serum for another 24h. As shown in Fig 3A (right part), PS exposure was reduced nearly 80% after 8h in normal serum (24h to 36h in Fig 3A). PS exposure then remained constant in the following 16h.

Confocal microscopy was used to visualize PS on the outer membrane of ECs. ECs were incubated with Alexa Fluor 647-CD31 and Alexa Fluor 488-lactadherin. As shown in Fig 3C, most ECs in uremic serum retracted from the cell-cell junctions, while the filopodia and localized regions on the cell margins stained with lactadherin. However, almost no staining by Alexa Fluor 488 -lactadherin could be detected on controls (Fig 3B).

### Procoagulant activity of blood cell, EC and MP in uremia

**Fig 4:**
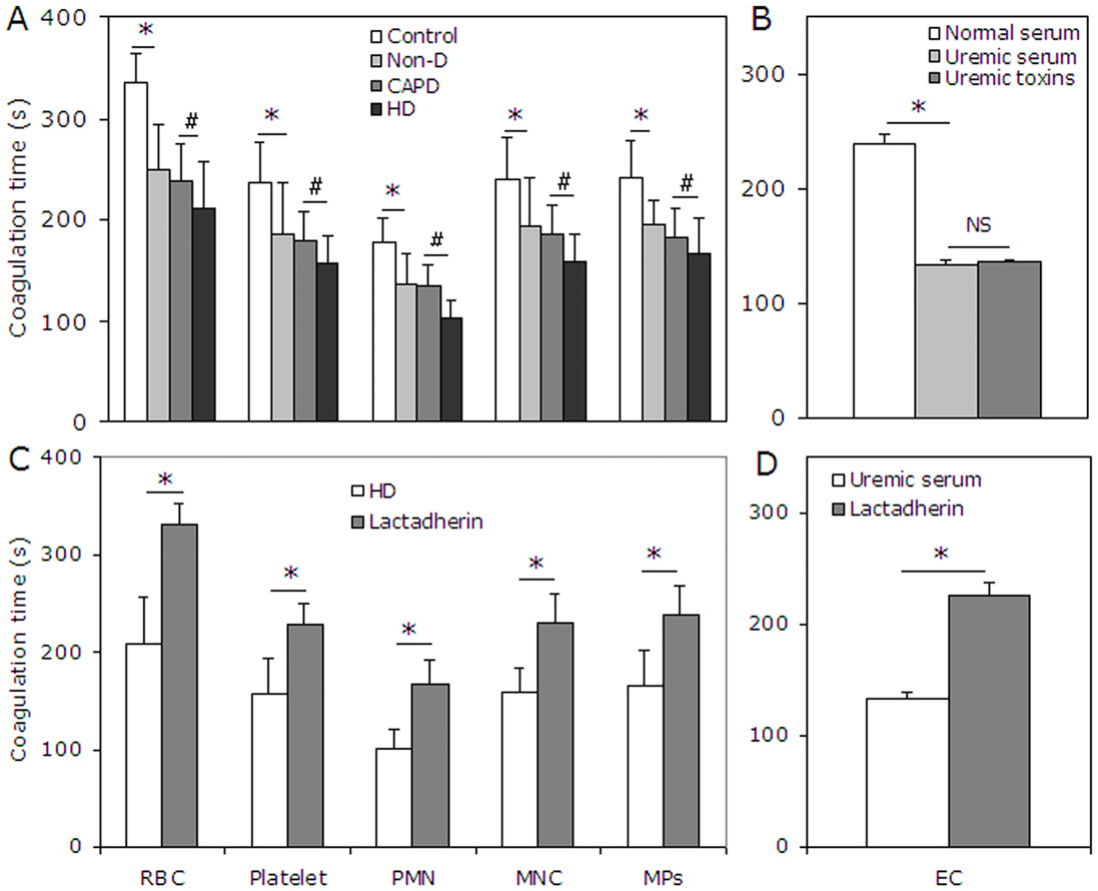
Coagulation time and inhibition assay. (**A**) Coagulation times of RBCs, platelets, PMNs, MNCs, and MPs from healthy subjects (n = 20), Non-D (n = 25), CAPD (n = 18) and HD patients (n = 23) were evaluated. Blood cells from uremic patient (especially HD) had more procoagulant activity than that from healthy subjects. (**B**) ECs cultured for 24 h in uremic serum or mixed toxins showed more procoagulant activity than those in normal serum. Data are representative of triplicate independent experiments. PCA of blood cell and MPs in 23 HD patients (**C**) and cultured ECs in uremic serum for 24h (**D**) was detected in the absence or presence of 128 nM lactadherin. Lactadherin almost entirely inhibited PCA supported by blood cell, EC and MP. Data are displayed as mean ± SD (*P < 0.001, ^#^P < 0.001). MPs: microparticles; PMN: polymorphomuclear cell; MNC: mononuclear cell; PCA: procoagulant activity; Non-D: Non-dialysis; CAPD: continuous ambulatory peritoneal dialysis; HD: haemodialysis.

**Fig 5:**
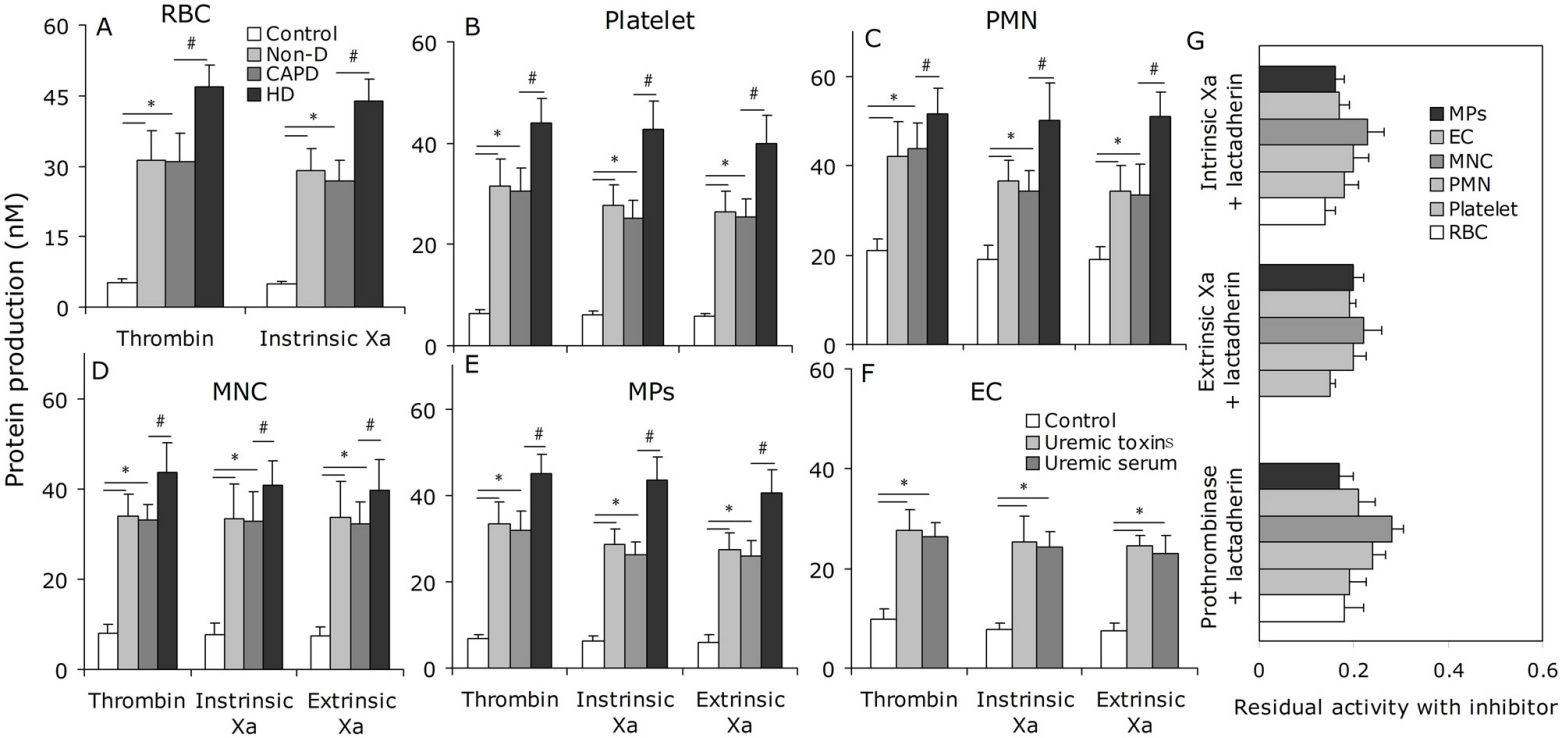
Formation and inhibition assays of procoagulant enzyme complexes. FXa and thrombin production of 2×10^5^ RBCs (**A**), 2×10^4^ platelets (**B**), 2×10^4^ PMN (**C**), 2×10^4^ MNC (**D**), 2×10^4^ ECs (**E**) and 10 μl-MPs from each group (20 healthy subjects, 25 Non-D, 18 CAPD and 23 HD) are shown. Intrinsic FXa formation was measured in the presence of FIXa, FVIII and thrombin. Extrinsic FXa production was assessed in the presence of FVIIa. Thrombin generation was investigated in the presence of FXa and FVa. Results displayed are mean ± SD (*P < 0.001, ^#^P < 0.001). (**F**) The capacity of 128 nM lactadherin to block procoagulant enzyme complexes on cells/MPs from 23 HD patients, and ECs incubated in uremic plasma was evaluated. In each kind of cell, lactadherin decreased activity of the procoagulant enzyme complexes by approximately 80%. Non-D: Non-dialysis; CAPD: continuous ambulatory peritoneal dialysis; HD: haemodialysis.

To investigate the relationship of PS externalization to hypercoagulable state in uremic patients, recalcification-time assays were used to assess the PCA of cultured ECs, blood cells and MPs from the study subjects. The results showed that the coagulation time was significantly reduced when using RBCs, platelets, PMNs, or MNCs from uremic patients (P < 0.001) compared with controls. Shorter coagulation times were also observed when using cells from HD versus CAPD or Non-D patients. Similar results were obtained using MPs (Fig 4A). ECs treated with uremic serum also showed increased PCA compared with controls (P < 0.001, Fig 4B). In order to explore the contribution of PS to the PCA of blood cells, ECs, and MPs in uremic patients, we performed coagulation inhibition assays. PCA of PBCs, ECs, and MPs was almost entirely inhibited by 128 nM lactadherin (Fig 4C and 4D). We further investigated the PCA of blood cells, ECs, and MPs using intrinsic, extrinsic FXa, and prothrombinase assays (Fig 5A–5F). The production of the three procoagulant enzyme complexes was increased in the uremic groups compared with controls (P < 0.001), and higher in HD than in CAPD and Non-D (P < 0.001 for each kind of cell). To determine the necessity of exposed PS on blood cells, ECs, or MPs to support procoagulant reactions, inhibition studies were also performed with lactadherin. For each kind of cell, 128 nM lactadherin blocked production of the three procoagulant enzyme complexes up to 80% (Fig 5G).

### FVa/FXa binding on MPs and cultured endothelial cell promote fibrin formation

**Fig 6:**
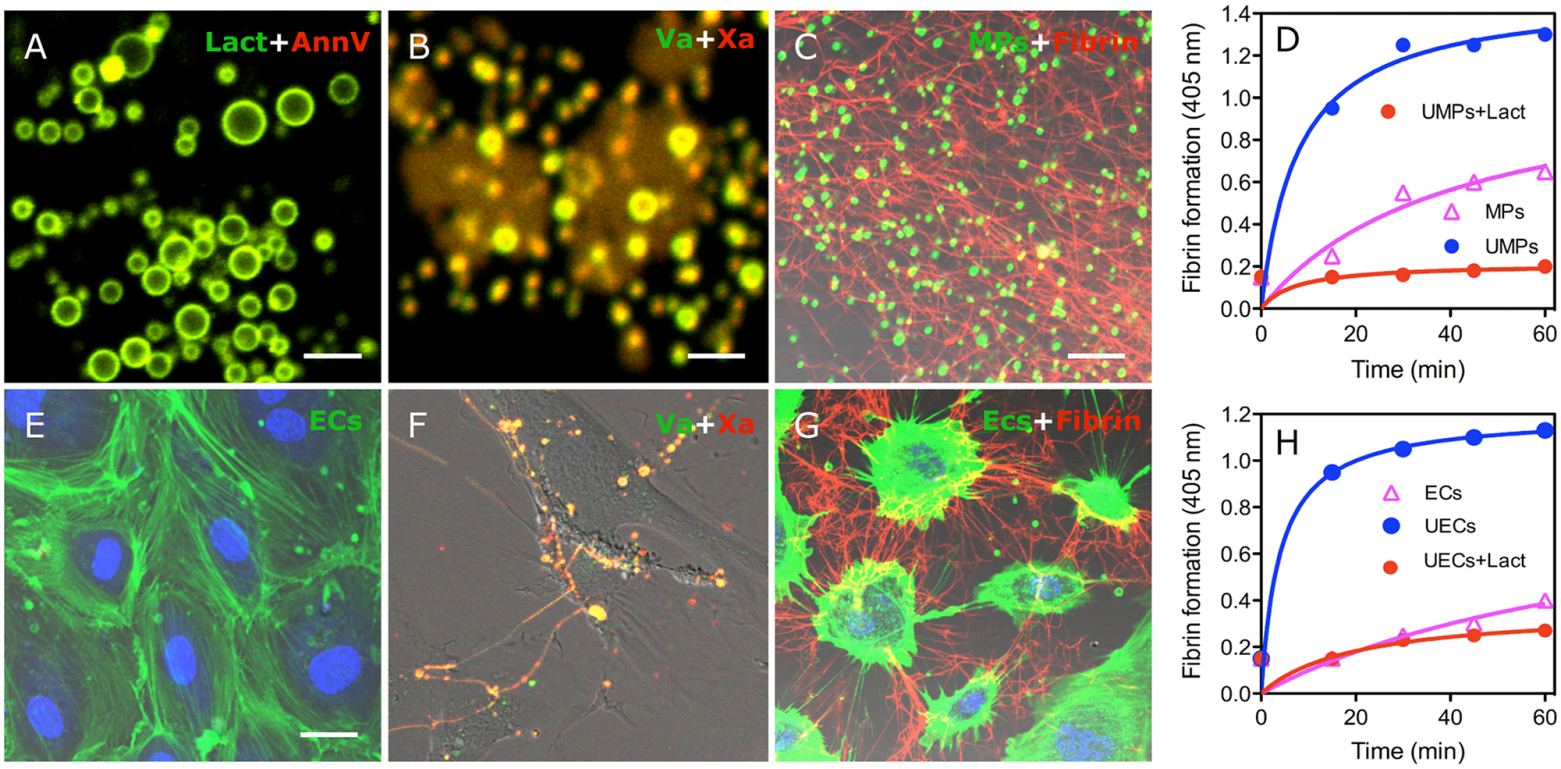
Circulating MPs from uremic patients and ECs treated with uremic serum support FVa/FXa binding and fibrin deposition. PS on circulating MPs, isolated from uremic patients, were co-stained (yellow) with lactadherin (green) and annexin V (red) **(A)** and co-bound with (yellow) factor Va-fluorescein-maleimide (green) and factor Xa-EGRck-biotin (red) **(B)** and visualized using confocal microscopy. **(C)** MPs were incubated with MDP in the presence of calcium for 30 min, stained with lactadherin (green), fixed, and stained with Alexa Fluro 647-anti-fibrin for 30 minutes. Converted fibrin networks were detected around uremic MPs. **(D)** Fibrin production, supported by normal-subject derived MPs (ΔMPs) and uremic patient derived MPs (UMPs), was measured by turbidity at 405 nm in the presence of recalcified MDP with or without 128 nM lactadherin or 25.6 μg/ml anti-TF using a SpectraMax 340PC plate reader. **(E)** ECs and nuclei were visualized by actin (green) and DAPI (blue). **(F)** FVa and FXa simultaneous staining (yellow) is observed on filopods near the retracted edges of uremic ECs similar to the binding sites for lactadherin. **(G)** Considerable fibrin was deposited radially along the filopodia between uremic ECs that were incubated with recalcified MDP for 30 min. **(H)** After adding recalcified MDP, dynamic fibrin formation on normal (ΔECs) and uremic serum cultured- ECs (UECs) was measured in the absence or presence of 128 nM lactadherin. Images were obtained by LSM 510 3.2 SP2 software. The inset bar equals 2 μm in A and B, 5 μm in C; 5 μm in E-G. PS: phosphatidylserine; MDP: MP-depleted plasma; UMPs: microparticles from uremic patient; ECs: Endothelial Cells; UECs: uremic serum cultured-EC.

We investigated the procoagulant role of PS on MPs from uremic patients and ECs cultured with uremic serum by FVa/FXa binding and fibrin formation assays using confocal microscopy. As shown in Fig 6A, MPs from uremic patient were costained with lactadherin and annexin V (yellow). Co-staining with factor Va-fluorescein-maleimide and factor Xa-EGRck-biotin (complexed to Alexa 647-steptavidin) indicated FVa (green) and FXa (red) have coherent distributions that overlap (yellow) around MPs (Fig 6B). As shown in Fig 6E, ECs with normal serum were visualized by actin and DAPI. FVa and Xa were enriched on the PS exposed sites of EC filopods after cultured with uremic serum at 24h, which support localized assembly of the prothrombinase complex on the margins of ECs and on filopodia that span the inter-cellular space (Fig 6F).

To observe fibrin formation, MPs of uremic patient were incubated with recalcified-MDP, resulting in the formation of a dense fibrin network among the MPs (Fig 6C). MDP was overlaid on washed ECs growing on a gelatin-coated chamber in the presence of calcium. Large fibrin strands were radially distributed along filopodia of uremic serum cultured- ECs (Fig 6G). We then used tubidity measurements to evaluate the ability of MPs and ECs to support fibrin formation. Uremic MPs or ECs significantly shortened the onset and increased the production of fibrin compared to healthy controls, respectively. This fibrin formation was blocked when lactadherin was included in the plasma (Fig 6D and 6H). These data indicate that uremic MPs and ECs trigger PS-dependent fibrin production.

## Discussion

Patients with uremia should be acknowledged as a group at high risk of cardiovascular events that require special attention [28]. In this study, we first showed that uremic patients live in a state of chronic PBC (including leukocyte, erythrocyte, platelet) and EC activation and/or injury, associated with increased MP release and PS exposure. Secondly, we showed that exposed PS on these cells provides binding sites for FVa and FXa, supports prothrombinase assembly, and promotes thrombin and fibrin generation. Lastly, we showed that blocking PS on PBCs, ECs, and MPs with lactadherin significantly inhibits activity of procoagulant enzyme complexes and consequently decreases PCA. The current findings lead us to conclude that these activated or injured cells may contribute to the thrombophilic tendency of uremia through PS exposure and MPs release.

We found that uremic patients, especially with HD, have a higher level of MPs compared with healthy subjects. In addition, we observed that RMP, PMP and LMP counts were significantly increased in HD patients when compared with CAPD and Non-D patients. These results may be due in part to haemodynamic changes during the HD procedure and repetitive mechanical stress on erythrocytes, platelets, and leukocytes, [29]. EMP counts were not affected by HD, but were correlated with creatinine levels, indicating a progressive decline in renal function. The exact mechanism of this correlation remains to be determined. Because MPs have been shown to act as indicators of cell activation or apoptosis, we measured the PS level on MP-derived cells. We found a significantly higher rate of PS exposure on PMN and MNC membranes in uremic patients especially in HD. This PS exposure appears to be related to the retention of uremic compounds and exposing blood to the less biocompatible dialysis membrane during the HD procedure which results in monocyte, neutrophil and lymphocyte activation or apoptosis [30–32]. In this study, the percentage RBCs and platelets exposing PS was increased in uremia. The absolute number of lactadherin^+^ RBC in uremic patients is higher than healthy subjects although total RBC counts are lower in uremic patients, which might be the result of cell activation, apoptosis, and/or elimination. In this study, PS exposure on PMPs and platelets was detected by staining with both annexin V and lactadherin. Consistent with the previous report [25], lactadherin exhibit higher PS sensitivity compared with annexin V, making it more suitable for PS detection.

Dysfunction of vascular endothelium is considered a critical event for the initiation of atherosclerosis [33], but there is conflicting data relating to the effect of uremic medium on EC proliferation and apoptosis [34–37]. We mimicked the *in vivo* conditions and evaluated the effect of uremic serum or mixed toxins on EC *in vitro*, and showed that these uremic toxins, similar to uremic serum, were able to induce PS externalization in ECs. Furthermore, when ECs were taken out of the uremic environment and reincubated in normal serum, exposed PS disappears in a large subpopulation of ECs, suggesting that ‘uremic toxins lesion’ of ECs in the early phase of renal failure is temporary and partly reversible. More importantly, we demonstrated that ECs treated with uremic serum bound FVa and FXa selectively on filopods and fibrils near the retracted edges of ECs. Further, fibrin deposition over EC filopods was observed by confocal microscopy. The fibrin formation on uremic ECs was shown to be PS dependent by the use of inhibitory lactadherin. These data showed that exposed PS on uremic EC surface provided membrane-binding sites for FVa/FXa and support for prothrombinase complex assembly, thrombin generation, and fibrin deposition. This data is consistent with Ocak G’s opinion that it might be endothelial damage associated with chronic kidney disease that leads to increased factor VIII and von Willebrand factor levels and eventually to venous thrombosis [7]. Our data suggests that PS is an important factor in this process. We examined only a subset of mixed uremic solutes that are known to have vascular effects rather than each kind of toxin, because our objective was to understand the procoagulant role of EC in the uremic milieu. Further studies are needed to elucidate the mechanism of toxin-mediated PS exposure.

Our present data suggest that the increased exposure of PS on the surface of PBCs, ECs, and MPs supports the assembly of prothrombinase and intrinsic/extrinsic factor Xase, which promotes the coagulation cascade reaction and subsequently leads to generation of thrombin and formation of fibrin. Excess thrombin formation contributes to hypercoagulability in uremia. Blockade of PS with lactadherin inhibited over 80% of procoagulant enzyme production and ultimately decreased the PCA of PBCs, ECs, and MPs. These results provide strong evidence that the PCA is mostly due to exposed PS on the surface of PBCs, ECs, and shed MPs. Our present study not only indicated increased PS exposure and MP release of blood cell and EC but also confirmed one mechanism inducing thromboembolic in uremia. Since toxins and HD are unavoidable in uremia, future studies into the possibility of using lactadherin in therapeutic strategies against uremic hypercoagulability, especially in accelerated atherosclerosis and stent thrombosis, are important.

In conclusion, our study systematically analyzes ECs and the various cells in peripheral blood, and shows that uremic toxins and HD induce a profoundly thrombogenic state mediated in large part by PS exposure and MPs release of PBC and EC. Additionally, PS appears to be a new marker or predictor of uremic thromboembolic complications.

## Supporting Information

S1 Fig Flow-cytometric analysis of MPs release and PS exposure in platelets. (TIF)

S1 Table Relationships between level of EMPs and the degree of renal insufficiency in 23 Non-D patients. (DOC)
