# Supplementary figures and images for: Thrombotic Role of Blood and Endothelial Cells in Uremia through Phosphatidylserine Exposure and Microparticle Release
Source: PLoS One. 2015 Nov 16;10(11):e0142835. doi: 10.1371/journal.pone.0142835 (PMC4646287; doi:10.1371/journal.pone.0142835)

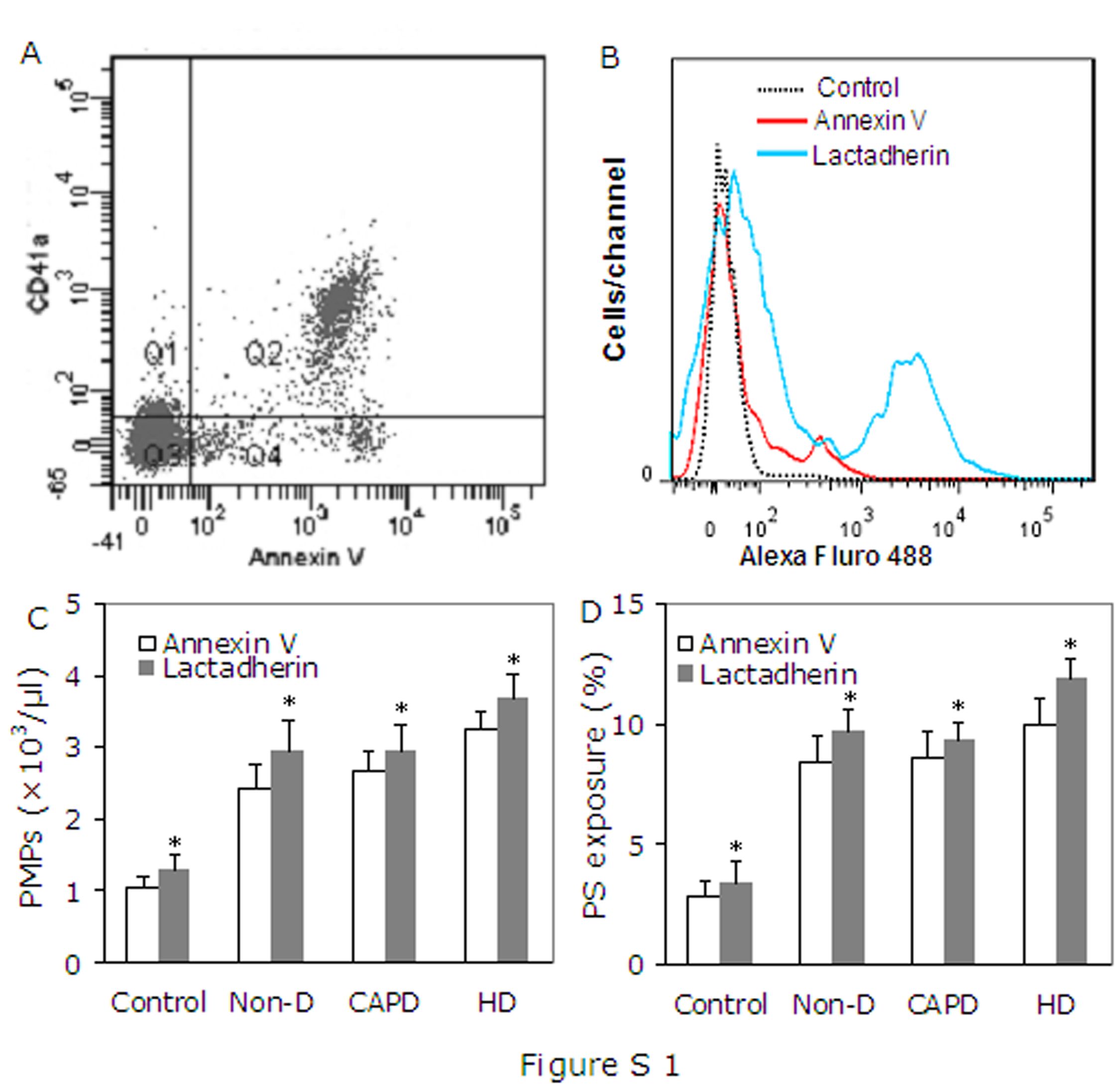

Supplement: S1 Fig — (TIF) [file pone.0142835.s001.tif]
