# Supplementary material for: Thrombotic Role of Blood and Endothelial Cells in Uremia through Phosphatidylserine Exposure and Microparticle Release
Source: PLoS One. 2015 Nov 16;10(11):e0142835. doi: 10.1371/journal.pone.0142835 (PMC4646287; doi:10.1371/journal.pone.0142835)
Supplement: S1 Table — (DOC) [file pone.0142835.s002.doc]

**S1 Table. Relationships between level of EMPs and the degree of renal insufficiency in 23 Non-D patients**

|  | *r* | *P*-value |
| --- | --- | --- |
| Creatinine | 0.527 | 0.017 |

Abbreviations: EMPs, endothelial cell MPs; Non-D, non-dialyzed. Values for *r* and *P* were obtained by the Pearson correlation test.
